# Supplementary material for: Cohort profile: Born in Wales—a birth cohort with maternity, parental and child data linkage for life course research in Wales, UK
Source: BMJ Open. 2024 Jan 18;14(1):e076711. doi: 10.1136/bmjopen-2023-076711 (PMC10806724; doi:10.1136/bmjopen-2023-076711)
Supplement: Supplementary data [file bmjopen-2023-076711supp001.pdf]

Supplementary Information

**Variable**	**N**	**%**
**Pregnancy Interval**		
Only/first child	345,645	49.85
Less than 1 year	6,234	0.90
1-2 years	73,319	10.57
2-5 years	176,109	25.40
5-7 years	45,141	6.51
7-10 years	29,868	4.31
Above 10 years	17,061	2.46
**Diabetes**		
No	692,777	99.91
Yes	600	0.09
**Anaemia**		
No	673,912	97.19
Yes	19,465	2.81
**Depression**		
No	684,553	98.73
Yes	8,824	1.27
**Serious mental illness**		
No	693,072	99.96
Yes	305	0.04
**Anxiety**		
No	682,812	98.48
Yes	10,565	1.52
**Antridepressant medication**		
No	693,213	99.98
Yes	164	0.02
**Smoking**		
No	544,258	78.49
Yes	149,119	21.51
**Alcohol-related hospital admission**		
No	692,709	99.90
Yes	668	0.10
**Substance misuse**		
No	658,520	94.97
Yes	34,857	5.03
**Domestic abuse**		
No	692,644	99.89
Yes	733	0.11
