# Supplementary material for: Cohort profile: Born in Wales—a birth cohort with maternity, parental and child data linkage for life course research in Wales, UK
Source: BMJ Open. 2024 Jan 18;14(1):e076711. doi: 10.1136/bmjopen-2023-076711 (PMC10806724; doi:10.1136/bmjopen-2023-076711)
Supplement: Author's manuscript [file bmjopen-2023-076711.draft_revisions.pdf]

BMJ Open

BMJ Open is committed to open peer review. As part of this commitment we make the peer review history of every article we publish publicly available.

When an article is published we post the peer reviewers' comments and the authors' responses online. We also post the versions of the paper that were used during peer review. These are the versions that the peer review comments apply to.

The versions of the paper that follow are the versions that were submitted during the peer review process. They are not the versions of record or the final published versions. They should not be cited or distributed as the published version of this manuscript.

BMJ Open is an open access journal and the full, final, typeset and author-corrected version of record of the manuscript is available on our site with no access controls, subscription charges or pay-per-view fees (http://bmjopen.bmj.com).

If you have any questions on BMJ Open's open peer review process please email info.bmjopen@bmj.com

BMJ Open

Cohort Profile: Born in Wales - a birth cohort with maternity, parental, and child data linkage for life course research in Wales, UK

Journal:	*BMJ Open*
Manuscript ID	bmjopen-2023-076711
Article Type:	Cohort profile
Date Submitted by the Author:	14-Jun-2023
Complete List of Authors:	Jones, Hope; Swansea University Medical School, National Centre for Population Health and Wellbeing Research<br>Seaborne, Michael; National Centre for Population Health and Wellbeing Research, Swansea University Medical School<br>Kennedy, Natasha; National Centre for Population Health and Wellbeing Research, Swansea University Medical School; Administrative Data Research Wales, Swansea University Medical School<br>James, M; National Centre for Population Health and Wellbeing Research, Swansea University Medical School<br>Dredge, Sam; National Centre for Population Health and Wellbeing research, Swansea University Medical School<br>Bandyopadhyay, Amrita; National Centre for Population Health and Wellbeing Research, Swansea University Medical School<br>Battaglia, Adele; Patient and Public Involvement Representative<br>Davies, Sarah; Betsi Cadwaladr University Health Board; Health and Care Research Wales<br>Brophy, Sinead; National Centre for Population Health and Wellbeing Research, Swansea University Medical School; Health Data Research UK
Keywords:	PUBLIC HEALTH, EPIDEMIOLOGY, Community child health < PAEDIATRICS

SCHOLARONE™
Manuscripts

I, the Submitting Author has the right to grant and does grant on behalf of all authors of the Work (as defined in the below author licence), an exclusive licence and/or a non-exclusive licence for contributions from authors who are: i) UK Crown employees; ii) where BMJ has agreed a CC-BY licence shall apply, and/or iii) in accordance with the terms applicable for US Federal Government officers or employees acting as part of their official duties; on a worldwide, perpetual, irrevocable, royalty-free basis to BMJ Publishing Group Ltd ("BMJ") its licensees and where the relevant Journal is co-owned by BMJ to the co-owners of the Journal, to publish the Work in this journal and any other BMJ products and to exploit all rights, as set out in our licence.

The Submitting Author accepts and understands that any supply made under these terms is made by BMJ to the Submitting Author unless you are acting as an employee on behalf of your employer or a postgraduate student of an affiliated institution which is paying any applicable article publishing charge ("APC") for Open Access articles. Where the Submitting Author wishes to make the Work available on an Open Access basis (and intends to pay the relevant APC), the terms of reuse of such Open Access shall be governed by a Creative Commons licence – details of these licences and which Creative Commons licence will apply to this Work are set out in our licence referred to above.

Other than as permitted in any relevant BMJ Author's Self Archiving Policies, I confirm this Work has not been accepted for publication elsewhere, is not being considered for publication elsewhere and does not duplicate material already published. I confirm all authors consent to publication of this Work and authorise the granting of this licence.

For peer review only

Cohort Profile: Born in Wales - a birth cohort with maternity, parental, and child data linkage for life course research in Wales, UK

Jones HE[1], Seaborne MJ[1,4], Kennedy NL[1,3], James ML[1], Dredge S[1], Bandyopadhyay A[1], Battaglia A[5], Davies S[6,7], Brophy S[1,2,3]

Affiliations:

[1]National Centre for Population Health and Wellbeing Research, Swansea University Medical School, Swansea, Wales, SA2 8PP, UK.

[2]Health Data Research UK,

[3]Administrative Data Research Wales, Swansea University Medical School, Swansea, Wales, SA2 8PP, UK.

[4]Data Lab, National Centre for Population Health and Wellbeing Research, Swansea University Medical School, Swansea, Wales, SA2 8PP, UK.

[5] Patient and Public Involvement Representative

[6] Betsi Cadwaladr University Health Board, Gwenfro, Wrexham Technology Park, Wrexham, LL13 7YP

[7] Health and Care Research Wales

Corresponding Author: h.e.jones@swansea.ac.uk

For peer review only

Abstract

Purpose: Parental and neonatal child health and education records have been linked to provide an entire country birth cohort, to examine what will improve the health and wellbeing of families growing up in Wales. Established in 2020, Born in Wales utilised data linkage techniques to connect information from the 2011 census with health, social care, and education routine data in the Secure Anonymised Information Linkage (SAIL) Databank. We present the descriptive data available in the linked database, emphasise the robust data security and governance frameworks, and present the future expansion plans for the database beyond its initial development stage.

Participants: Descriptive information from 2011 to 2023 has been gathered from SAIL. This comprehensive dataset comprises over 400,000 child electronic records. To augment this data, the Born in Wales and primary school surveys have contributed quantitative and qualitative responses.

Findings to date: The cohort comprises all children born in Wales since 2011, with follow-up conducted until they finish primary school at age 11. 2,500 parents and 30,000 primary school children have been recruited for enhanced data collection and linkage to the data spine. The child cohort is 51%: 49% female: male, and 6% are from ethnic minority backgrounds. When considering age distribution, 26.8% of children are under the age of 5, while 63.2% fall within the age range of 5-11.

Future plans: Born in Wales will expand by 30,000 new births annually in Wales, while including follow-up data of children and parents already in the database. Supplementary datasets complement the existing linkage, including primary care, hospital data, educational attainment and social care. Future research includes exploring the long-term implications of COVID-19 on

For peer review only

child health and development, the influence of environmental factors including climate change on health and examining the impact of parental work environment on child health and development.

For peer review only

For peer review only - http://bmjopen.bmj.com/site/about/guidelines.xhtml

Strengths and limitations of this study

- Born in Wales has established a comprehensive, Wales-wide population-based database which consolidates clinical data from maternity, neonatal, child health, and education records.
- This national-scale database is supplemented by quantitative and qualitative results from surveys conducted by Born in Wales, providing rich insights into details that cannot be obtained through routinely collected data.
- The existence of this database enables further data linkage, facilitating life course research on the health and wellbeing of the Wales population.
- Missing data or errors in routine and administrative data may be constraint.
- A potential restriction of Born in Wales is the loss of data pertaining to individuals who relocate outside of Wales during pregnancy or after the child's birth.

For peer review only

Introduction

The recognition of health promotion early in life is acknowledged as a strategy to enhance health and wellbeing across the life course [1]. This longitudinal approach encompasses the stages of pregnancy planning, pregnancy, childhood, adolescence, and adulthood [2]. Birth cohorts such as Born in Bradford (BiB) [3], the eLIXIR Partnership [4], and Born in Scotland [5] have contributed substantial evidence in the field of life course research, particularly focusing on pregnancy and early childhood.

Traditional cohorts are formed by recruiting participants within a specified timeframe. However, these cohorts can very quickly become outdated due to fluctuations in population demographics, lifestyle factors, and environmental variations. Follow-up of such cohorts becomes challenging, as sample attrition, where participants withdraw, leave, or do not complete the study, introduces methodological biases that can impact research validity and findings [6]. Furthermore, cohort recruitment may suffer from volunteer bias or self-selection bias, potentially limiting the representativeness of the cohort [7]. Population-based cohorts eliminate these biases and provide broader information about a wider scale of outcomes that may not be feasible in other cohort studies [8].

Previous birth cohorts have faced challenges related to attrition and inclusion bias. Routine electronic health records, which encompass data on all women receiving antenatal care in Wales and their infants, offer a solution to reduce these complications [9]. While national birth cohorts have been well established in other countries [10,11], the linkage of maternal and child data has not been extensively utilised in the UK. In Scotland, population data linkage has yielded findings that have informed UK clinical guidelines regarding maternal and neonatal outcomes [12].

For peer review only - http://bmjopen.bmj.com/site/about/guidelines.xhtml

Numerous successful linkages, such as maternity data with national birth registration datasets, birth registration with Hospital Episode Statistics (HES), and utilisation of primary care pregnancy data in the UK [13-17], have demonstrated the utility of this approach.

Born in Wales facilitates a platform for linking research datasets with authorised levels of patient anonymity and assured data security. Additional advantages include the capability to consolidate various datasets from other organisations, including healthcare, social care, and education. Ethical guidelines must be adhered to when working with these datasets to ensure accurate data linkage and participant anonymity.

Data-linkage facilitated by Born in Wales provides a unique data repository for investigating significant public health questions with far-reaching implications. The capacity to run these linkages permits a wide range of longitudinal health, social, and education data to be collected along with the ability to enhance life course data analysis. Born in Wales operates as a longitudinal database, commencing in pregnancy, and incorporating routinely collected clinical data from maternity, neonatal, social care, and education records, not solely relying on participant recruitment. Moreover, Born in Wales has the added element of supplementation by survey responses as well as the population-level data outcomes. With records of over 30,000 new births in Wales annually, Born in Wales has the potential to evolve into one of the most comprehensive datasets on parental and child health. The Secure Anonymised Information Linkage (SAIL) Databank [18,19] plays a vital role in integrating and harmonising these datasets.

This document outlines the safeguards implemented to protect user rights throughout the development and application of Born in Wales, as well as providing the demographics of the

For peer review only

Born in Wales cohort. These procedures and mechanisms adopted are established on the successes observed in other databases and cohorts [3-5].

For peer review only

For peer review only - http://bmjopen.bmj.com/site/about/guidelines.xhtml

Cohort description

Data sources

Born in Wales establishes linkages between electronic data pertaining to mothers, babies, and partners (including self-identified biological fathers or mothers' partners) for children born in Wales, encompassing approximately 30,000 annual births. The linked health records encompass primary care data (from Wales Longitudinal General Practice (WLGP)), secondary care (from hospital admissions, emergency care, inpatient from Patient Episode Database for Wales (PEDW), and outpatient from Outpatient Database for Wales (OPDW)), maternal indicators (midwife data), public health records (vaccination uptake, hearing checks, health visitor assessment, breastfeeding initiation and duration, and COVID-19 vaccination/testing) as well as a core database for the National Community Child Health Database (NCCH). Additionally, data pertaining to education, Census 2011, police/domestic violence, substance abuse, social care (looked after children, child protection register, children in receipt of care, and family court) have been successfully linked. Moreover, connections are being established to Census 2021 and the National Neonatal Audit Database. Ultrasound scans conducted across Wales have been coded to identify follow-up markers predictive of future cognitive development and school readiness, these have also been prepared for integration into the Born in Wales data spine. Maternity and neonatal data were acquired from the SAIL Databank, extracting from multiple datasets including the NCCH Dataset, Maternal Indicators Dataset (MIDS), and Patient Episode Dataset for Wales (PEDW). Please refer to Table 1 for further details regarding the datasets already linked or planned to be linked in Born in Wales.

For peer review only - http://bmjopen.bmj.com/site/about/guidelines.xhtml

In addition to the electronic birth cohort, the data spine is enhanced with repeated surveys administered to both mothers and identified biological fathers or female/male partners during pregnancy. These surveys capture self-reported health data pertaining to stress, mental health, occupation, ethnicity, and open ended questions addressing strategies to enhance health and wellbeing for families [20]. Over a 5 year period, an average of 1,000 women/families per year will be targeted for recruitment to further enrich the birth cohort. The enhanced data obtained from these surveys will be utilised to extrapolate (impute data) for the larger cohort study, enabling the estimation of variables such as income in the comprehensive all Wales cohort. It is important to note that 94% of the population in Wales is of Caucasian ethnicity.

**Datasets**	**Example of data fields**
**Annual District Birth Extract (ADBE)**	Register of all births and stillbirths (cause of death) in Wales, multiple births, Lower layer Super Output Area (LSOA) of mother, place of birth, no father on registration, marriage status of parents, 10% fathers' socio-economic status.
**Congenital Anomaly Register and Information Service (CARS)**	Congenital anomaly (ICD10 code), gender, LSOA, information about mother including substance abuse, migrant status, diabetes, epilepsy.
**Education Wales (EDUW)**	Attainment (foundation phase, key stage 1 and 2), free school meal, attendance, special educational needs, education other than school, ethnicity, age, gender, local

For peer review only - http://bmjopen.bmj.com/site/about/guidelines.xhtml

	authority.
**Patient Episode Dataset for Wales (PEDW)**	Admission to hospital diagnosis and operation codes
**Outpatient Dataset for Wales (OPDW)**	Attendance information for all NHS Wales hospital outpatient appointments
**Maternal Indicators Dataset (MIDS)**	Maternal data at initial assessment including age, smoking, weight, mental health condition, previous births, Labour and birth data including Apgar Score, birth outcome (c-section, assisted delivery, presentation e.g., transverse), birth weight, intention to breastfeed.
**National Community Child Health Database (NCCH)**	Breastfeeding duration, birth weight, gestation, blood test results, maternal smoking, hearing/vision tests, Health visitor assessment of family resilience, domestic violence, speech and language skills, flying start services.
**ONS 2011 Census Wales (CENW) (2021 Census to be linked)**	Ethnicity, Household composition, occupation, qualifications, place of work, long term health problem or disability, house data (number of rooms), gender, LSOA.
**Children Receiving Care and**	On child protection register, abuse type, disability,

For peer review only

For peer review only - http://bmjopen.bmj.com/site/about/guidelines.xhtml

**Support Census (CRCS)**	ethnicity, autism, parental capacity, dental health, mental health,
**Substance Misuse Dataset (SMDS)**	Gender, age, substance abuse problem, parental responsibility, accommodation, mental health.
**Children and Family Court Advisory and Support Service (CAFCASS)**	Looks after the interests of children involved in family proceedings. Works under the rules of the Family Court and legislation to work with children and their families, then advise the courts on what is in the best interests of individual children.
**Welsh Longitudinal General Practice Dataset (WLGP) - Welsh Primary Care**	Diagnosis, medications, symptoms and procedures.
**Welsh Demographic Service Dataset (WDSD)**	People registered with a GP in Wales, gender, LSOA, deprivation score, Residential Linking Field, Date left Wales.
**Geographic mapping**	Environmental data including distance to facilities
**Born in Wales (BIW) surveys (Expectant Parent, 18–24-month, Nursery)**	Wellbeing, stress, physical activity, ethnicity, nationality, relationship status, sexual orientation, mood/depression, alcohol consumption, smoking, weight/height, maternity care, breastfeeding, local area

For peer review only

	services, occupation, education, income, brushing teeth, child activity and development.
**Health & Attainment of Pupils in Primary EducatioN (HAPPEN)**	Wellbeing, physical activity, diet, Me and My Feelings questionnaire (behavioural difficulty, emotional difficulty), garden, safety of area, hours of sleep, teeth brushing, ability to swim, ability to ride a bike.
**Welsh Study of Mothers and Babies (WOMBS)**	Ultrasound scan for cognitive development
**COVID-19 Vaccination and Testing**	COVID-19 vaccination uptake rates and tests

Table 1. Datasets linked or to be linked in Born in Wales

Data-linkage hosting environment

The core datasets of Born in Wales consist of PEDW, MIDS, and NCCH. These datasets are enhanced by linking them to HAPPEN, CENW, WOMBS, and Born in Wales survey data. Further linkages then can include multiple datasets including CARS, EDUW, CAFCASS and CRCS. The core components of Born in Wales and subsequent linkages are described in Figure 1. The foundation of Born in Wales involves SAIL's robust anonymising system, which ensures secure data linkage in adherence to specified data protocols. As part of the data upload process to SAIL, the original dataset is divided into two distinct types of files. File 1 encompasses sensitive person-level demographics data, which is transmitted to Digital Health and Care Wales (DHCW). DHCW undertakes the processing, matching, and anonymisation of the File 1 data

For peer review only - http://bmjopen.bmj.com/site/about/guidelines.xhtml

before transmitting it to SAIL. File 2 comprises clinical data or other non-identifiable data, which is directly transmitted to SAIL (See flow diagram of this process in Figure 2).

Figure 1. Core components of Born in Wales and further linkages

Figure 2. Flow diagram of the SAIL process

Data-linkage procedures and resources

SAIL employs an Anonymous Linking Field (ALF) to establish connections with other records. Matching of two records arises when their respective ALFs are identical. Within the Born in Wales database, both maternal and child ALFs are utilised, enabling the identification and linkage of mothers to their corresponding children. Consequently, this integration culminates in the creation of a comprehensive data repository, accessible to researchers who can request extracted data encompassing maternity, neonatal, and/or mental health services. The matching algorithm was collaboratively designed and assessed by a trustworthy third party and SAIL. The algorithm compares numerous personal identifiers between the received dataset and the Welsh Demographic Service Dataset (WDSD). The linkage is performed based on an NHS number whenever possible (deterministic linkage). In the absence of an NHS number, a matching algorithm utilising surname, first name, post code, date of birth and sex is applied against the Welsh Demographic Service (WDS) Dataset (probabilistic linkage). The algorithm's development ensures a high matching accuracy, with specific thresholds for match accuracy reported to SAIL within the anonymised dataset. A poor matching score indicates incomplete or inaccurate personal information provided by an individual or a lack of registration on the NHS database. The ALF system ensures a high level of match quality, as it facilitates the accurate matching of infants born within Born in Wales with their respective mothers' records [18].

For peer review only - http://bmjopen.bmj.com/site/about/guidelines.xhtml

Patient and public involvement

Patient and Public Involvement (PPI) is integral to the growth of Born in Wales, underscoring its commitment to inclusivity and participatory research. The inclusion of PPI ensures that members of the public actively participate in shaping the research project. Valuable input was sought during the design of the questionnaires, complemented by the insights provided by midwives who shared their lived experiences on pertinent research inquiries. Moreover, a dedicated PPI steering group will be actively sought to incorporate feedback from the public. The PPI group will be recruited from midwives and health visitors via already established networks, along with expectant and new parents to help co-produce its delivery and approach from the survey to research questions and aims. Born in Wales aims to adopt a co-production approach, emphasising collaborative partnerships among researchers, practitioners, and the public. The decision-making processes within Born in Wales will prioritise PPI to ensure the inclusion of diverse perspectives. To facilitate this approach, Born in Wales will follow the Co-production of Research and Strategy (CORDS) standard operating procedure [21] and the UK standards for PPI involvement and guidance from INVOLVE [22] which establishes a framework for meaningful engagement with practitioners and families. This approach fosters a reciprocal exchange, focusing on the important questions and concerns for stakeholders, while also enhancing the impact, translation, and dissemination of findings. A record of PPI activity will be maintained using the Public Involvement in Research Impact Toolkit (PIRIT) [23].

For peer review only - http://bmjopen.bmj.com/site/about/guidelines.xhtml

Findings to date

Born in Wales cohort characteristics

The cohort comprises all children born in Wales since 2011 and followed until age 11. An electronic data spine has been established to encompass the entire population of children born in Wales in the last 13 years, resulting in a cohort size exceeding 400,000 children within Born in Wales. Furthermore, 2,500 parents and 30,000 primary school children have been recruited to facilitate enhanced data collection, all of whom are linked to the electronic cohort data spine; ongoing recruitment efforts are underway. This cohort encompasses the entirety of Wales, thus represents a national birth cohort for the country. In terms of gender distribution, females comprise 51% of the cohort, while males account for 49%, additionally, 6% of the cohort are from an ethnic minority background. Analysis of the NCCH Births dataset reveals that 26.8% of children are below the age of 5, whereas 63.2% are aged between 5-11 years. Between 2011 to 2022, a total of N=435,032 births were recorded, involving N=269,427 mothers. Of these births, N=222,867 are male, N=212,188 are female births, and N=40 the gender data is missing.

Maternal, birth and birth outcomes

Between 2011 to 2022, there were N=1,718 stillbirths, constituting 0.4% of all births. Examining gestational age, 7.26% (N=31,625) of infants were born preterm, defined as delivery before 37 weeks gestation, while 82.8% (N=360,825) were born at term or later (37 weeks and onwards). The Born in Wales database has recently been utilised to investigate the risk factors associated with low birth weight (LBW). Analyses revealed that non-singleton children exhibited the highest risk of LBW (OR 21.74 (95% CI 21.09 to 22.40)), followed by pregnancy intervals of less than 1 year (OR 2.92 (95% CI 2.70 to 3.15)). Maternal physical and mental health

For peer review only - http://bmjopen.bmj.com/site/about/guidelines.xhtml

conditions, including diabetes (OR 2.03 (95% CI 1.81 to 2.28)), anaemia (OR 1.26 (95% CI 1.16 to 1.36)), depression (OR 1.58 (95% CI 1.43 to 1.75)), serious mental illness (OR 1.46 (95% CI 1.04 to 2.05)), anxiety (OR 1.22 (95% CI 1.08 to 1.38)) and use of antidepressant medication during pregnancy (OR 1.92 (95% CI 1.20 to 3.07)) were also identified as significant risk factors for LBW. Additional maternal risk factors include smoking (OR 1.80 (95% CI 1.76 to 1.84)), alcohol-related hospital admission (OR 1.60 (95% CI 1.30 to 1.97)), substance misuse (OR 1.35 (95% CI 1.29 to 1.41)) and evidence of domestic abuse (OR 1.98 (95% CI 1.39 to 2.81)) [24].

Born in Wales survey participants

Among the participants of the Born in Wales survey, the mean age was 32 years (SD=12.5), with an interquartile range (IQR) of 28-35 years. The majority of survey participants identified as white ethnicity, accounting for 45.5% of the sample (51% did not disclose their ethnicity). The Born in Wales survey data was utilised in a mixed methods study conducted during the COVID-19 pandemic, aiming to examine the effects of the pandemic on pregnancy experiences and birth outcomes in 2020. Results indicated that the pandemic had a notable adverse effect on the psychological wellbeing of 71% of survey respondents. These individuals reported heightened levels of anxiety, stress, and feelings of loneliness, which were associated with attending prenatal scans in the absence of their partner, giving birth alone, and restricted interactions with midwives. However, no significant differences were observed in annual birth outcomes, including gestation, birth weight, stillbirths, and Caesarean sections, between infants born in 2020 compared to 2016–2019. Whilst the pandemic negatively affected mothers' pregnancy experiences, population-level data indicates that this did not result in adverse birth outcomes for infants born during the pandemic [25].

For peer review only

For peer review only - http://bmjopen.bmj.com/site/about/guidelines.xhtml

HAPPEN children's primary school network

Health & Attainment of Pupils in Primary EducatioN (HAPPEN) represents a substantial cohort of over 30,000 school-aged children across Wales, with a gender distribution of approximately 47% boys, 49% girls and 3% who preferred not to disclose their gender. The average age of the children is 9.35 years. Recently, the HAPPEN dataset has been utilised to explore the health and wellbeing of children during the COVID-19 pandemic. This encompassed a retrospective cohort study employing an online cohort survey conducted between January 2018 to February 2020, in conjunction with routine PCR SARS-CoV-2 test results. The study explored health-related behaviours in children spanning the period from 2018 to 2020, and their association with being tested and testing positive in 2020 to 2021. Notably, the investigation revealed significant associations between parental health literacy and monitoring behaviours [26]. Furthermore, the study employed free school meal status (FSM) as a proxy for deprivation in an exploratory analysis examining the impact of school closures on the health and wellbeing. The findings indicated that children eligible for FSM may experience adverse consequences in terms of physical health, including reduced physical activity and suboptimal dietary choices, as a result of prolonged school closures [27].

For peer review only

Discussion

Strengths and limitations

Born in Wales has established a comprehensive, Wales-wide population-based database which consolidates clinical data from maternity, neonatal, child health, and education records. This national-scale database is supplemented by quantitative and qualitative results from surveys conducted by Born in Wales, providing rich insights into details that cannot be obtained through routinely-collected data. The existence of this database enables further data linkage, facilitating life course research on the health and wellbeing of the Wales population. This has significant implications for enhancing healthcare delivery at the local level and offering valuable research evidence to guide policy and practice both within the studied population and in other countries. This research aims to generate robust evidence that can influence policy and practice, enabling the implementation of early interventions to promote child health and wellbeing. Moreover, collaborations with diverse cohorts facilitate comparisons across different populations, enabling a broader understanding of factors influencing child health and wellbeing outcomes.

One inherent constraint associated with routine and administrative data is the presence of missing data or errors. However, SAIL has established a robust framework that includes a team of skilled analysts and rigorous quality control procedures to address issues such as duplication of patient data entries and minimise instances of missing data. Subsequently, the Born in Wales project can contribute to the enhancement of clinical reporting practice and bolster the reliability of research findings derived from the database. Additionally, the utilisation of anonymised cohorts serves as an effective strategy for overcoming the barriers related to obtaining consent from individuals, thereby facilitating the seamless aggregation and analysis of data.

For peer review only - http://bmjopen.bmj.com/site/about/guidelines.xhtml

A potential constraint of Born in Wales is the loss of data pertaining to individuals who relocate outside of Wales during pregnancy or after the child's birth. Analogous to other datasets, research conducted utilising Born in Wales may be constrained by the information available through routine data collection. Nevertheless, Born in Wales can enhance the available information by incorporating survey questions that capture data missing from administrative records. It is important to acknowledge that data entry can be subject to human error, and there may still be instances of missing records.

Research aspirations

As the Born in Wales database continues to expand, there are intentions to broaden the scope by incorporating additional health, social care, and education data, alongside Police data. The subsequent phase of data linkage will involve integrating Police data into SAIL. Replicating this data linkage model on a national scale within the UK and potentially extending it globally would enable the establishment of more extensive research cohorts and facilitate cross-comparisons across diverse populations.

This cohort is established through the integration of routine data records, encompassing health and administrative data, as well as environmental data supplemented with survey data to enrich the cohort. It constitutes a comprehensive, data rich total population cohort constructed through substantial investments and extensive record linkage efforts from HDR UK and ADR. Furthermore, this methodology demonstrates cost-effectiveness by leveraging existing data sources, establishing a dynamic cohort that continuously expands its participant pool. The cohort is well suited for investigating natural experiments and interventions. The endeavours undertaken

For peer review only

in this study closely align with recommendations 1-3 and 6 of the MRC strategy to maximise UK population cohorts [28].

The design of this cohort facilitates cross-cohort comparisons with other electronic cohorts in the UK, including Born in Bradford [3] and eLIXIR [4]. To harmonise phenotypic variables across studies and adopt core common data standards, all data in the cohort will adhere to the OMOP standard. Knowledge sharing and directories have been facilitated through the HDR UK gateway, and high-quality, robust meta-data is provided, adhering to recommendations 4 and 5 of the MRC strategy [28].

This cohort is unique due to the comprehensive breadth of routine and survey data collected and linked in a total population cohort. It is designed to enable data sharing by employing harmonised data and meta-data, following the principles of findability, accessibility, interoperability, and reusability (FAIR). A distinguishing feature of Born in Wales is its incorporation of census data linked to numerous datasets, encompassing social care and justice datasets. Additionally, the database includes self-reported data which captures aspects that are not typically electronically captured, such as stress, wellbeing, physical activity, access to services, and use of services.

Data availability and access

The data for the Born in Wales cohort is available in the SAIL databank at Swansea University, Swansea, UK [29]. All proposals to use SAIL data are subject to review by an independent Information Governance Review Panel (IGRP). This project's approval code is 0916. Before any data can be accessed, approval must be given by the IGRP. To use Born in Wales data you need

For peer review only

For peer review only - http://bmjopen.bmj.com/site/about/guidelines.xhtml

to provide a safe researcher training certificate, a signed data access agreement and IGRP approval.

Future directions

A comprehensive research database has been established, encompassing data pertaining to maternity, neonatal, and child health. This database integrates information on maternal and paternal health throughout pregnancy and extends to encompass the subsequent health of the child. Moreover, it enriches the dataset with valuable qualitative insights obtained from the Born in Wales surveys, which capture nuanced information not captured solely by hospital records. Born in Wales is committed to continuous progress and expansion through collaborative efforts, enabling longitudinal follow-up of families in Wales and fostering co-production of research questions relevant to each stage of development. By encompassing the entire population of Wales, Born in Wales generates a wealth of rich data on the national scale, which has the potential to inform interventions aimed at promoting healthy growth and development among the population.

For peer review only

Data availability statement: Data are available upon reasonable request. Researchers can apply for data access by submitting a research application to the SAIL team. The SAIL website provides information on the application process (https://saildatabank.com/data/apply-to-work-with-the-data/). All proposals to use SAIL data are subject to review by an independent Information Governance Review Panel (IGRP). This project's approval code is 0916. Before any data can be accessed, approval must be given by the IGRP. To use Born in Wales data you need to provide a safe researcher training certificate, a signed data access agreement and IGRP approval.

Funding: This work was supported by the National Centre for Population Health and Wellbeing Research (NCPHWR) grant number [AMS103836].

Contributors: HJ, SB and SD contributed to the conception of this article. MS, NK, MJ, AB, AB, and SD were involved in manuscript writing and revision. HJ, MS, AB, and MJ were involved in data analysis and interpretation. All authors read and approved the final manuscript.

Patient and public involvement: Patients and/or the public were involved in the design and dissemination of this research and a PPI steering group is being sought.

For peer review only

References

[1] O'Connor M, Spry E, Patton G et al. Better together: Advancing life course research through multi-cohort analytic approaches. *Adv. Life Course Res*. 2022;53:100499.

[2] Mikkelsen B, Williams J, Rakovac I et al. Life course approach to prevention and control of non-communicable diseases. *BMJ* [Internet]. 2019;364:l257. Available from: https://www.bmj.com/content/364/bmj.l257

[3] Wright J, Small N, Raynor P et al. Cohort Profile: The Born in Bradford multi-ethnic family cohort study. *Int. J. Epidemiol.* [Internet]. 2012 Oct 12 [cited 2019];42(4):978–91. Available from: https://academic.oup.com/ije/article/42/4/978/655870

[4] Carson LE, Azmi B, Jewell A et al. Cohort profile: the eLIXIR Partnership—a maternity–child data linkage for life course research in South London, UK. *BMJ Open*. 2020;10(10):e039583.

[5] Born in Scotland [Internet]. The University of Edinburgh. 2022 [cited 2023 Jun 14]. Available from: https://www.ed.ac.uk/cardiovascular-science/born-in-scotland

[6] Li M, Wang Z, Zhang B et al. Sample attrition analysis in a prospective cohort study of medical graduates in China. *BMC Med. Res. Methodol.* 2022;22(1).

[7] Ferri-García R, Rueda M del M. Propensity score adjustment using machine learning classification algorithms to control selection bias in online surveys. Marchetti S, editor. *PLoS One*. 2020;15(4):e0231500.

For peer review only - http://bmjopen.bmj.com/site/about/guidelines.xhtml

[8] Our Research [Internet]. HDR UK. Available from: https://www.hdruk.ac.uk/research/

[9] Gray L. The importance of post hoc approaches for overcoming non-response and attrition bias in population-sampled studies. *Soc. Psychiatry Psychiatr. Epidemiol*. 2015;51(1):155–7.

[10] Todberg T, Koch A, Andersson M et al. Incidence of Otitis Media in a Contemporary Danish National Birth Cohort. Schooling CM, editor. *PLoS One*. 2014;9(12):e111732.

[11] Brown AS, Sourander A, Hinkka-Yli-Salomäki S et al . Elevated maternal C-reactive protein and autism in a national birth cohort. *Mol. Psychiatry* [Internet]. 2014 Feb 1 [cited 2022];19(2):259–64. Available from: https://pubmed.ncbi.nlm.nih.gov/23337946/

[12] Norman JE, Morris C, Chalmers JD. The Effect of Changing Patterns of Obstetric Care in Scotland (1980–2004) on Rates of Preterm Birth and Its Neonatal Consequences: Perinatal Database Study. *PLoS Med*. 2009;6(9):e1000153–3.

[13] Martin P, Cortina-Borja M, Newburn M et al. Timing of singleton births by onset of labour and mode of birth in NHS maternity units in England, 2005–2014: A study of linked birth registration, birth notification, and hospital episode data. Dangal G, editor. *PLoS One*. 2018;13(6):e0198183.

[14] Thornton CE, Dahlen HG. Born before arrival in NSW, Australia (2000–2011): a linked population data study of incidence, location, associated factors and maternal and neonatal outcomes. *BMJ Open* [Internet]. 2018;8(3):e019328. Available from: https://bmjopen.bmj.com/content/bmjopen/8/3/e019328.full.pdf

For peer review only - http://bmjopen.bmj.com/site/about/guidelines.xhtml

[15] Harper G. Linkage of Maternity Hospital Episode Statistics data to birth registration and notification records for births in England 2005–2014: Quality assurance of linkage of routine data for singleton and multiple births. *BMJ Open*. 2018;8(3):e017898.

[16] Dattani N, Macfarlane A. Linkage of Maternity Hospital Episode Statistics data to birth registration and notification records for births in England 2005-2014: methods. A population-based birth cohort study. *BMJ Open*. 2018;8(2):e017897.

[17] Birth cohort data linkage study [Internet]. ADR UK. [cited 2023 Jun 14]. Available from: https://www.adruk.org/our-mission/our-impact/article/birth-cohort-data-linkage-study-228/

[18] Jones KH, Ford DV, Thompson S et al. A Profile of the SAIL Databank on the UK Secure Research Platform. *IJPDS*. 2020;4(2).

[19] Lyons RA, Ford DV, Moore L et al. Use of data linkage to measure the population health effect of non-health-care interventions. *Lancet*. 2014;383(9927):1517–9.

[20] Born in Wales [Internet]. NCPHWR. [cited 2023 Jun 14]. Available from: https://ncphwr.org.uk/portfolio/born-in-wales-overview/

[21] CORDS (Co-production of Research Direction and Strategy) [Internet]. NCPHWR. [cited 2023 Jun 14]. Available from: https://ncphwr.org.uk/portfolio/cords-co-production/

[22] Guidance on co-producing a research project – INVOLVE [Internet]. Available from: https://www.invo.org.uk/posttypepublication/guidance-on-co-producing-a-research-project/

For peer review only

[23] Public Involvement in Research Impact Toolkit (PIRIT) [Internet]. Cardiff University. Available from: https://www.cardiff.ac.uk/marie-curie-research-centre/patient-and-public-involvement/public-involvement-in-research-impact-toolkit-pirit

[24] Bandyopadhyay A, Jones H, Parker M et al. Weighting of risk factors for low birth weight: a linked routine data cohort study in Wales, UK. *BMJ Open*. 2023;13(2):e063836–6.

[25] Jones H, Seaborne M, Cowley L et al. Population birth outcomes in 2020 and experiences of expectant mothers during the COVID-19 pandemic: A "born in Wales" mixed methods study using routine data. Magnus MC, editor. *PLoS One*. 2022;17(5):e0267176

[26] Marchant E, Lowthian E, Crick T et al. Pre-COVID-19 pandemic health-related behaviours in children (2018–2020) and association with being tested for SARS-CoV-2 and testing positive for SARS-CoV-2 (2020–2021): a retrospective cohort study using survey data linked with routine health data in Wales, UK. *BMJ Open*. 2022;12(9):e061344–4.

[27] James M, Marchant E, Defeyter MA et al. Impact of school closures on the health and well-being of primary school children in Wales UK: a routine data linkage study using the HAPPEN Survey (2018–2020). *BMJ Open*. 2021;11(10):e051574.

[28] Maximising the value of UK population cohorts MRC strategic Review of the largest UK Population cohort studies [Internet]. [cited 2023 Jun 14]. Available from: https://www.ukri.org/wp-content/uploads/2021/12/MRC-23122021-UK-Population-cohorts.pdf

[29] Ford DV, Jones KH, Verplancke JP, et al. The SAIL Databank: building a national architecture for e-health research and evaluation. *BMC Health Serv. Res*. 2009;9(1)

For peer review only - http://bmjopen.bmj.com/site/about/guidelines.xhtml

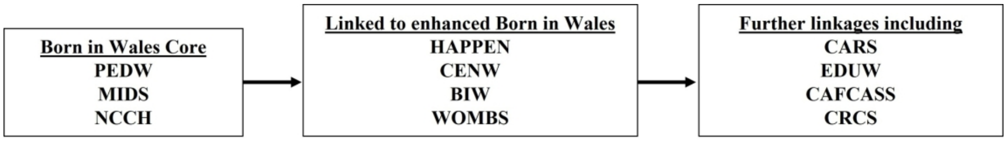

Figure 1. Core components of Born in Wales and further linkages

177x24mm (300 x 300 DPI)

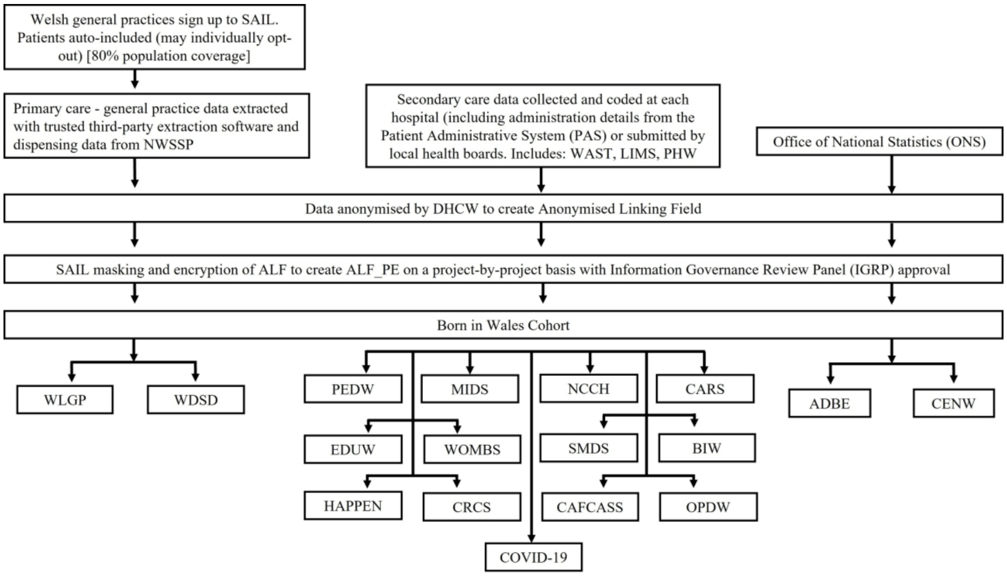

Figure 2. Flow diagram of the SAIL process

231x132mm (300 x 300 DPI)

STROBE 2007 (v4) Statement—Checklist of items that should be included in reports of *cohort studies*

Section/Topic	Item #	Recommendation	Reported on page #
**Title and abstract**	1	(*a*) Indicate the study's design with a commonly used term in the title or the abstract	1
		(*b*) Provide in the abstract an informative and balanced summary of what was done and what was found	1-2
**Introduction**			
Background/rationale	2	Explain the scientific background and rationale for the investigation being reported	4-5
Objectives	3	State specific objectives, including any prespecified hypotheses	5
**Methods**			
Study design	4	Present key elements of study design early in the paper	5
Setting	5	Describe the setting, locations, and relevant dates, including periods of recruitment, exposure, follow-up, and data collection	8
Participants	6	(*a*) Give the eligibility criteria, and the sources and methods of selection of participants. Describe methods of follow-up	7
		(*b*) For matched studies, give matching criteria and number of exposed and unexposed	NA
Variables	7	Clearly define all outcomes, exposures, predictors, potential confounders, and effect modifiers. Give diagnostic criteria, if applicable	NA
Data sources/ measurement	8*	For each variable of interest, give sources of data and details of methods of assessment (measurement). Describe comparability of assessment methods if there is more than one group	*7-8*
Bias	9	Describe any efforts to address potential sources of bias	4
Study size	10	Explain how the study size was arrived at	14
Quantitative variables	11	Explain how quantitative variables were handled in the analyses. If applicable, describe which groupings were chosen and why	14-15
Statistical methods	12	(*a*) Describe all statistical methods, including those used to control for confounding	NA
		(*b*) Describe any methods used to examine subgroups and interactions	14-15
		(*c*) Explain how missing data were addressed	NA
		(*d*) If applicable, explain how loss to follow-up was addressed	NA
		(*e*) Describe any sensitivity analyses	NA
**Results**			

For peer review only

Participants	13*	(a) Report numbers of individuals at each stage of study—eg numbers potentially eligible, examined for eligibility, confirmed eligible, included in the study, completing follow-up, and analysed	NA
		(b) Give reasons for non-participation at each stage	NA
		(c) Consider use of a flow diagram	12
Descriptive data	14*	(a) Give characteristics of study participants (eg demographic, clinical, social) and information on exposures and potential confounders	14
		(b) Indicate number of participants with missing data for each variable of interest	NA
		(c) Summarise follow-up time (eg, average and total amount)	20
Outcome data	15*	Report numbers of outcome events or summary measures over time	15-18
Main results	16	(*a*) Give unadjusted estimates and, if applicable, confounder-adjusted estimates and their precision (eg, 95% confidence interval). Make clear which confounders were adjusted for and why they were included	NA
		(*b*) Report category boundaries when continuous variables were categorized	NA
		(*c*) If relevant, consider translating estimates of relative risk into absolute risk for a meaningful time period	NA
Other analyses	17	Report other analyses done—eg analyses of subgroups and interactions, and sensitivity analyses	15-17
**Discussion**			
Key results	18	Summarise key results with reference to study objectives	18
**Limitations**			
Interpretation	20	Give a cautious overall interpretation of results considering objectives, limitations, multiplicity of analyses, results from similar studies, and other relevant evidence	18
Generalisability	21	Discuss the generalisability (external validity) of the study results	19-20
**Other information**			
Funding	22	Give the source of funding and the role of the funders for the present study and, if applicable, for the original study on which the present article is based	22

*Give information separately for cases and controls in case-control studies and, if applicable, for exposed and unexposed groups in cohort and cross-sectional studies.

Note: An Explanation and Elaboration article discusses each checklist item and gives methodological background and published examples of transparent reporting. The STROBE checklist is best used in conjunction with this article (freely available on the Web sites of PLoS Medicine at http://www.plosmedicine.org/, Annals of Internal Medicine at http://www.annals.org/, and Epidemiology at http://www.epidem.com/). Information on the STROBE Initiative is available at www.strobe-statement.org.

For peer review only

BMJ Open

Cohort Profile: Born in Wales - a birth cohort with maternity, parental, and child data linkage for life course research in Wales, UK

Journal:	*BMJ Open*
Manuscript ID	bmjopen-2023-076711.R1
Article Type:	Cohort profile
Date Submitted by the Author:	13-Dec-2023
Complete List of Authors:	Jones, Hope; National Centre for Population Health and Wellbeing Research, Swansea University Medical School<br>Seaborne, Michael; National Centre for Population Health and Wellbeing Research, Swansea University Medical School<br>Kennedy, Natasha; National Centre for Population Health and Wellbeing Research, Swansea University Medical School; Administrative Data Research Wales, Swansea University Medical School<br>James, M; National Centre for Population Health and Wellbeing Research, Swansea University Medical School<br>Dredge, Sam; National Centre for Population Health and Wellbeing research, Swansea University Medical School<br>Bandyopadhyay, Amrita; National Centre for Population Health and Wellbeing Research, Swansea University Medical School<br>Battaglia, Adele; Patient and Public Involvement Representative<br>Davies, Sarah; Betsi Cadwaladr University Health Board; Health and Care Research Wales<br>Brophy, Sinead; National Centre for Population Health and Wellbeing Research, Swansea University Medical School; Health Data Research UK
**Primary Subject Heading**:	Public health
Secondary Subject Heading:	Paediatrics
Keywords:	PUBLIC HEALTH, EPIDEMIOLOGY, Community child health < PAEDIATRICS, Surveys and Questionnaires

SCHOLARONE™
Manuscripts

I, the Submitting Author has the right to grant and does grant on behalf of all authors of the Work (as defined in the below author licence), an exclusive licence and/or a non-exclusive licence for contributions from authors who are: i) UK Crown employees; ii) where BMJ has agreed a CC-BY licence shall apply, and/or iii) in accordance with the terms applicable for US Federal Government officers or employees acting as part of their official duties; on a worldwide, perpetual, irrevocable, royalty-free basis to BMJ Publishing Group Ltd ("BMJ") its licensees and where the relevant Journal is co-owned by BMJ to the co-owners of the Journal, to publish the Work in this journal and any other BMJ products and to exploit all rights, as set out in our licence.

The Submitting Author accepts and understands that any supply made under these terms is made by BMJ to the Submitting Author unless you are acting as an employee on behalf of your employer or a postgraduate student of an affiliated institution which is paying any applicable article publishing charge ("APC") for Open Access articles. Where the Submitting Author wishes to make the Work available on an Open Access basis (and intends to pay the relevant APC), the terms of reuse of such Open Access shall be governed by a Creative Commons licence – details of these licences and which Creative Commons licence will apply to this Work are set out in our licence referred to above.

Other than as permitted in any relevant BMJ Author's Self Archiving Policies, I confirm this Work has not been accepted for publication elsewhere, is not being considered for publication elsewhere and does not duplicate material already published. I confirm all authors consent to publication of this Work and authorise the granting of this licence.

For peer review only

Cohort Profile: Born in Wales - a birth cohort with maternity, parental, and child data linkage for life course research in Wales, UK

Jones HE[1], Seaborne MJ[1,4], Kennedy NL[1,3], James ML[1], Dredge S[1], Bandyopadhyay A[1], Battaglia A[5], Davies S[6,7], Brophy S[1,2,3]

Affiliations:

[1]National Centre for Population Health and Wellbeing Research, Swansea University Medical School, Swansea, Wales, SA2 8PP, UK.

[2]Health Data Research UK, Swansea University Medical School, Swansea, Wales, SA2 8PP, UK.

[3]Administrative Data Research Wales, Swansea University Medical School, Swansea, Wales, SA2 8PP, UK.

[4]Data Lab, National Centre for Population Health and Wellbeing Research, Swansea University Medical School, Swansea, Wales, SA2 8PP, UK.

[5] Patient and Public Involvement Representative

For peer review only

For peer review only - http://bmjopen.bmj.com/site/about/guidelines.xhtml

[6] Betsi Cadwaladr University Health Board, Gwenfro, Wrexham Technology Park, Wrexham, LL13 7YP

[7] Health and Care Research Wales

Corresponding Author: h.e.jones@swansea.ac.uk

For peer review only

For peer review only - http://bmjopen.bmj.com/site/about/guidelines.xhtml

Abstract

Purpose: Using Wales's national dataset for maternity and births as a core dataset, we have linked related datasets to create a more complete and comprehensive entire country birth cohort. Data of anonymised identified persons are linked on the individual level to data from health, social care, and education data within the Secure Anonymised Information Linkage (SAIL) Databank. Each individual is assigned an encrypted Anonymised Linking Field (ALF); this field is used to link anonymised individuals across datasets. We present the descriptive data available in the core dataset, and the future expansion plans for the database beyond its initial development stage.

Participants: Descriptive information from 2011 to 2023 has been gathered from the National Community Child Health Database (NCCHD) in SAIL. This comprehensive dataset comprises over 400,000 child electronic records. Additionally, survey responses about health and wellbeing from a cross-section of the population including 2,500 parents and 30,000 primary school children have been collected for enriched personal responses and linkage to the data spine.

Findings to date: The electronic cohort comprises all children born in Wales since 2011, with follow-up conducted until they finish primary school at age 11. The child cohort is 51%: 49% female: male, and 7.8% are from ethnic minority backgrounds. When considering age distribution, 26.8% of children are under the age of 5, while 63.2% fall within the age range of 5-11.

Future plans: Born in Wales will expand by 30,000 new births annually in Wales (in NCCHD), while including follow-up data of children and parents already in the database. Supplementary datasets complement the existing linkage, including primary care, hospital data, educational

For peer review only - http://bmjopen.bmj.com/site/about/guidelines.xhtml

attainment, and social care. Future research includes exploring the long-term implications of COVID-19 on child health and development, and examining the impact of parental work environment on child health and development.

For peer review only

For peer review only - http://bmjopen.bmj.com/site/about/guidelines.xhtml

Strengths and limitations of this study

- Born in Wales has established a comprehensive, Wales-wide population-based database which consolidates clinical data from maternity, neonatal, child health, and education records.
- This national-scale database is enriched by quantitative and qualitative results from surveys conducted by Born in Wales, providing rich insights into details that cannot be obtained through routinely collected data.
- The existence of this database enables further data linkage, facilitating life course research on the health and wellbeing of the Wales population.
- Missing data or errors in routine and administrative data may be a constraint.
- A potential restriction of Born in Wales is the loss of data pertaining to individuals who relocate outside of Wales during pregnancy or after the child's birth.

For peer review only - http://bmjopen.bmj.com/site/about/guidelines.xhtml

Introduction

The recognition of health promotion early in life is acknowledged as a strategy to enhance health and wellbeing across the life course [1]. This longitudinal approach encompasses the stages of pregnancy planning, pregnancy, childhood, adolescence, and adulthood [2]. Birth cohorts such as Born in Bradford (BiB) [3], the eLIXIR Partnership [4], and Born in Scotland [5] have contributed substantial evidence in the field of life course research, particularly focusing on pregnancy and early childhood.

Traditional cohorts are formed by recruiting participants within a specified timeframe. However, these cohorts can very quickly become outdated due to fluctuations in population demographics, lifestyle factors, and environmental variations. Follow-up of such cohorts becomes challenging, as sample attrition, where participants withdraw, leave, or do not complete the study, introduces methodological biases that can impact research validity and findings [6]. Furthermore, cohort recruitment may suffer from volunteer bias or self-selection bias, potentially limiting the representativeness of the cohort [7]. Population-based cohorts eliminate these biases and provide broader information about a wider scale of outcomes that may not be feasible in other cohort studies [8].

Previous birth cohorts have faced challenges related to attrition and inclusion bias. Routine electronic health records, which encompass data on all women receiving antenatal care in Wales and their infants, offer a solution to reduce these complications [9]. While national birth cohorts have been well established in other countries [10,11], the linkage of maternal and child data has not been extensively utilised in the UK. In Scotland, population data linkage has yielded findings that have informed UK clinical guidelines regarding maternal and neonatal outcomes [12].

For peer review only - http://bmjopen.bmj.com/site/about/guidelines.xhtml

Numerous successful linkages, such as maternity data with national birth registration datasets, birth registration with Hospital Episode Statistics (HES), and utilisation of primary care pregnancy data in the UK [13-17], have demonstrated the utility of this approach.

Born in Wales facilitates a platform for linking research datasets with authorised levels of patient anonymity and assured data security. Additional advantages include the capability to consolidate various datasets from other organisations, including healthcare, social care, and education. Ethical guidelines must be adhered to when working with these datasets to ensure accurate data linkage and participant anonymity.

The Secure Anonymised Information Linkage (SAIL) Databank [18,19] plays a vital role in integrating and harmonising these datasets. This document outlines the safeguards implemented to protect user rights throughout the development and application of Born in Wales, as well as providing the demographics of the Born in Wales cohort. These procedures and mechanisms are established based on the successes observed in other databases and cohorts [3-5].

Data-linkage facilitated by Born in Wales provides a unique data repository for investigating significant public health questions with far-reaching implications. The capacity to run these linkages permits a wide range of longitudinal health, social, and education data to be collected along with the ability to enhance life course data analysis. Born in Wales operates as a longitudinal database, commencing in pregnancy, and incorporating routinely collected clinical data from maternity, neonatal, social care, and education records, not solely relying on participant recruitment. Moreover, Born in Wales has the added element of enrichment by survey responses as well as the population-level data outcomes.

For peer review only - http://bmjopen.bmj.com/site/about/guidelines.xhtml

The core dataset for Born in Wales is the National Community Child Health Database (NCCHD) which provides child birth information, such as birth weight, gestation time, birth order, as well as information regarding the mother (e.g. smoker, type of maternity care). This database is updated quarterly. With over 30,000 annual new births in Wales, Born in Wales has the potential to evolve into one of the most comprehensive datasets on parental and child health.

This document outlines how we have used the NCCHD as a core dataset to build a more comprehensive and complete maternity and birth cohort in Wales which can be linked and used for future longitudinal life-course research analyses.

For peer review only

Cohort description

Data sources

Born in Wales establishes linkages between electronic data pertaining to mothers, babies, and partners, including self-identified biological fathers or mothers' partners, for children born in Wales, encompassing approximately 30,000 annual births. The core dataset is NCCHD, which can be linked to various other records. The linked health records encompass primary care data (from Wales Longitudinal General Practice (WLGP)), secondary care (from hospital admissions, emergency care, inpatient from Patient Episode Database for Wales (PEDW), and outpatient from Outpatient Database for Wales (OPDW)), maternal indicators (midwife data), and public health records (vaccination uptake, hearing checks, health visitor assessment, breastfeeding initiation and duration, and COVID-19 vaccination/testing). Additionally, data pertaining to education, Census 2011, police/domestic violence, substance abuse, social care (looked after children, child protection register, children in receipt of care, and family court) have been successfully linked. Moreover, connections are being established to Census 2021 and the National Neonatal Audit (NNAP) database. Ultrasound scans conducted across Wales have been coded to identify follow-up markers predictive of future cognitive development and school readiness; these have also been prepared for integration into the Born in Wales data spine.

Maternity and neonatal data were acquired from the SAIL Databank, extracting from multiple datasets including the NCCHD, Maternal Indicators Dataset (MIDS), and PEDW. Please refer to Table 1 for further details regarding the datasets already linked or planned to be linked in Born in Wales.

For peer review only - http://bmjopen.bmj.com/site/about/guidelines.xhtml

In addition to the electronic birth cohort, the data spine is enriched with repeated surveys administered to both mothers and identified biological fathers or female/male partners during pregnancy, and children in primary school. These surveys capture self-reported health data pertaining to stress, mental health, occupation, ethnicity, and open ended questions addressing strategies to enhance health and wellbeing for families [20]. Since 2016, 30,000 primary school children have completed the Health & Attainment of Pupils in Primary EducatioN (HAPPEN) Survey. Primary schools are recruited via e-mail and local authority referral. The survey is completed online, in school, at a time that suits the school. The survey has been developed alongside the new school curriculum for Wales priorities for health and wellbeing. Since 2020, 2,500 parents have been recruited via social media to complete the Born in Wales surveys. This survey is also completed online. For both surveys, participants provide informed consent to be included in the cohort, and recruitment is ongoing. Over a 5 year period, we aim for an average of 1,000 women/families per year to complete the surveys to further enrich the birth cohort. The data obtained from these surveys will be utilised to extrapolate (impute data) for the larger cohort study, enabling the estimation of variables such as income in the comprehensive all Wales cohort.

**Datasets**	**Example of data fields**
**Electronic Data**	
**National Community Child Health Database (NCCHD)**	Breastfeeding duration, birth weight, gestation, blood test results, maternal smoking, hearing/vision tests, Health visitor assessment of family resilience, domestic violence, speech and language skills, flying start services.

For peer review only - http://bmjopen.bmj.com/site/about/guidelines.xhtml

**Annual District Birth Extract (ADBE)**	Register of all births and stillbirths (cause of death) in Wales, multiple births, Lower layer Super Output Area (LSOA) of mother, place of birth, no father on registration, marriage status of parents, 10% fathers' socio-economic status.
**Congenital Anomaly Register and Information Service (CARS)**	Congenital anomaly (ICD10 code), gender, LSOA, information about mother including substance abuse, migrant status, diabetes, epilepsy.
**Education Wales (EDUW)**	Attainment (foundation phase, key stage 1 and 2), free school meal, attendance, special educational needs, education other than school, ethnicity, age, gender, local authority.
**Patient Episode Dataset for Wales (PEDW)**	Admission to hospital diagnosis and operation codes
**Outpatient Dataset for Wales (OPDW)**	Attendance information for all NHS Wales hospital outpatient appointments
**Maternal Indicators Dataset (MIDS)**	Maternal data at initial assessment including age, smoking, weight, mental health condition, previous births, Labour and birth data including Apgar Score, birth outcome (c-section, assisted delivery, presentation e.g., transverse), birth weight, intention to breastfeed.
**ONS 2011 Census Wales (CENW) (2021 Census to be linked)**	Ethnicity, Household composition, occupation, qualifications, place of work, long term health problem or disability, house data (number of rooms), gender, LSOA.
**Children Receiving Care and Support Census (CRCS)**	On child protection register, abuse type, disability, ethnicity, autism, parental capacity, dental health, mental health,
**Substance Misuse Dataset (SMDS)**	Gender, age, substance abuse problem, parental responsibility, accommodation, mental health.
**Children and Family Court Advisory and Support Service (CAFCASS)**	Looks after the interests of children involved in family proceedings. Works under the rules of the Family Court and legislation to work with children and their families, then advise the courts on what is in the best interests of individual children.
**Welsh Longitudinal General Practice Dataset (WLGP) - Welsh Primary Care**	Diagnosis, medications, symptoms and procedures.
**Welsh Demographic Service Dataset (WDSD)**	People registered with a GP in Wales, gender, LSOA, deprivation score, Residential Linking Field, Date left Wales.

For peer review only

For peer review only - http://bmjopen.bmj.com/site/about/guidelines.xhtml

**Geographic mapping**	Environmental data including distance to facilities
**Welsh Study of Mothers and Babies (WOMBS)**	Ultrasound scan for cognitive development
**COVID-19 Vaccination and Testing**	COVID-19 vaccination uptake rates and tests
**Survey Data**	
**Born in Wales (BIW) surveys (Expectant Parent, 18–24-month, Nursery)**	Wellbeing, stress, physical activity, ethnicity, nationality, relationship status, sexual orientation, mood/depression, alcohol consumption, smoking, weight/height, maternity care, breastfeeding, local area services, occupation, education, income, brushing teeth, child activity and development.
**Health & Attainment of Pupils in Primary EducatioN (HAPPEN)**	Wellbeing, physical activity, diet, Me and My Feelings questionnaire (behavioural difficulty, emotional difficulty), garden, safety of area, hours of sleep, teeth brushing, ability to swim, ability to ride a bike.

Table 1. Datasets linked or to be linked in Born in Wales

Data-linkage hosting environment

The core datasets of Born in Wales consist of NCCHD, PEDW, and MIDS. These datasets are enhanced by linking them to CENW, WOMBS, and the survey data from Born in Wales and HAPPEN. Further linkages can include multiple datasets including CARS, EDUW, CAFCASS, and CRCS. The core components of Born in Wales and subsequent linkages are described in Figure 1. The foundation of Born in Wales involves SAIL's robust anonymising system, which ensures secure data linkage in adherence to specified data protocols. As part of the data upload process to SAIL, the original dataset is divided into two distinct types of files. File 1 encompasses sensitive person-level demographics data, which is transmitted to Digital Health and Care Wales (DHCW). DHCW undertakes the processing, matching, and anonymisation of

For peer review only - http://bmjopen.bmj.com/site/about/guidelines.xhtml

the File 1 data before transmitting it to SAIL. File 2 comprises clinical data or other non-identifiable data, which is directly transmitted to SAIL (See flow diagram of this process in Figure 2).

Figure 1. Core components of Born in Wales and further linkages

Figure 2. Flow diagram of the SAIL process

Data-linkage procedures and resources

SAIL employs an Anonymous Linking Field (ALF) to establish connections with other records. Matching of two records arises when their respective ALFs are identical. Within the Born in Wales database, both maternal and child ALFs are utilised, enabling the identification and linkage of mothers to their corresponding children. Consequently, this integration culminates in the creation of a comprehensive data repository, accessible to researchers who can request extracted data encompassing maternity, neonatal, and/or mental health services. The matching algorithm was collaboratively designed and assessed by a trustworthy third party and SAIL. The algorithm compares numerous personal identifiers between the received dataset and the Welsh Demographic Service Dataset (WDSD). The linkage is performed based on an NHS number whenever possible (deterministic linkage). In the absence of an NHS number, a matching algorithm utilising surname, first name, post code, date of birth, and sex is applied against the WDSD (probabilistic linkage). The algorithm's development ensures a high matching accuracy, with specific thresholds for match accuracy reported to SAIL within the anonymised dataset. A poor matching score indicates incomplete or inaccurate personal information provided by an individual or a lack of registration on the NHS database. The ALF system ensures a high level of

For peer review only - http://bmjopen.bmj.com/site/about/guidelines.xhtml

match quality, as it facilitates the accurate matching of infants born within Born in Wales with their respective mothers' records [18].

Patient and public involvement

Patient and Public Involvement (PPI) is integral to the growth of Born in Wales, underscoring its commitment to inclusivity and participatory research. The inclusion of PPI ensures that members of the public actively participate in shaping the research project. Valuable input was sought during the design of the surveys, complemented by insights from midwives who shared their lived experiences on pertinent research inquiries. Moreover, a dedicated PPI steering group will be actively sought to incorporate feedback from the public. The PPI group will be recruited from midwives and health visitors via already established networks, along with expectant and new parents to help co-produce its delivery and approach from the survey to research questions and aims. Born in Wales aims to adopt a co-production approach, emphasising collaborative partnerships among researchers, practitioners, and the public. The decision-making processes within Born in Wales will prioritise PPI to ensure the inclusion of diverse perspectives. To facilitate this approach, Born in Wales will follow the Co-production of Research and Strategy (CORDS) standard operating procedure [21] and the UK standards for PPI involvement and guidance from INVOLVE [22] which establishes a framework for meaningful engagement with practitioners and families. This approach fosters a reciprocal exchange, focusing on the important questions and concerns for stakeholders, while also enhancing the impact, translation, and dissemination of findings. A record of PPI activity will be maintained using the Public Involvement in Research Impact Toolkit (PIRIT) [23].

Ethical Statement

For peer review only - http://bmjopen.bmj.com/site/about/guidelines.xhtml

This study was approved by the SAIL Databank independent Information Governance Review Panel (IGRP project number 0916, Wales Electronic Cohort for Children Phase 4). The survey elements were approved by the Health Research Authority (protocol number RIO 030-20).

For peer review only

For peer review only - http://bmjopen.bmj.com/site/about/guidelines.xhtml

Findings to date

Born in Wales cohort characteristics

The cohort comprises all children born in Wales since 2011 (from NCCHD) and followed until age 11. An electronic data spine has been established to encompass the entire population of children born in Wales in the last 13 years, resulting in a cohort size exceeding 400,000 children within Born in Wales. Furthermore, 2,500 parents and 30,000 primary school children have been recruited to facilitate enriched data collection, all of whom are linked to the electronic cohort data spine. The Born in Wales electronic cohort encompasses the entirety of Wales, thus represents a national birth cohort for the country. In terms of gender distribution, females comprise 51% of the cohort, while males account for 49%. 78.5% of the population in Wales is of Caucasian ethnicity with only 7.8% of the cohort from an ethnic minority background. Analysis of the NCCHD Births dataset reveals that 26.8% of children are below the age of 5, whereas 63.2% are aged between 5-11 years. Between 2011 to 2022, a total of N=435,031 births were recorded, involving N=269,421 mothers. Of these births, N=222,814 are male, N=212,198 are female births, and N=19 gender data is missing. Table 2 represents the characteristics of the electronic cohort.

**Characteristic**	**Value**	**N**	**Percentage (%)**
**Mothers**	Mothers	269,421	100
**Babies**	Babies	435,031	100
**Sex (of baby)**	Male	222,814	51.22
	Female	212,198	48.78
	Missing	19	0.00

For peer review only - http://bmjopen.bmj.com/site/about/guidelines.xhtml

**Ethnic group (of mother)**	White - any background	211,728	78.49
	Gypsy or Irish Traveller	23	0.01
	White & Black Caribbean	883	0.33
	White & Black African	1,153	0.43
	White & Asian	1,153	0.43
	Any other mixed background / multiple ethnic background	3,209	1.19
	Indian	1,922	0.71
	Pakistani	1,750	0.65
	Bangladeshi	1,439	0.53
	Chinese	2,284	0.85
	Any other Asian background	262	0.10
	Caribbean	2,156	0.80
	African	568	0.21
	Any other Black background	793	0.29
	Any other ethnic group	3,396	1.26
	Arab	14	0.01
	Missing	37,026	13.73
**Stillbirths**	Live Birth	433,315	99.61
	Stillbirth	1,716	0.39
**Gestation age category**	Extremely premature (EPT)	2,278	0.52

For peer review only - http://bmjopen.bmj.com/site/about/guidelines.xhtml

	Very premature (VPT)	3,552	0.82
	Premature (PT)	25,809	5.93
	Term (T)	345,277	79.37
	Late term (LT)	15,738	3.62
	Very late term (VLT)	285	0.07
	Missing	42,092	9.68
**Birth weight category**	Extremely low birth weight (ELBW)	35,333	8.12
	Very low birth weight (VLBW)	2,249	0.52
	Low birth weight (LBW)	21,179	4.87
	Normal birth weight (NBW)	296,585	68.18
	High birth weight (HBW)	36,877	8.48
	Very high birth weight (VHBW)	5,897	1.36
	Missing	36,911	8.48
**Singleton or multiple birth**	Multiple	11,656	2.68
	Singleton	408,456	93.89
	Missing	14,919	3.43

Table 2. Characteristics of the cohort

Maternal, birth and birth outcomes

Between 2011 to 2022, there were N=1,716 stillbirths, constituting 0.39% of all births.

Examining gestational age, 7.27% (N=31,639) of infants were born preterm, defined as delivery

For peer review only - http://bmjopen.bmj.com/site/about/guidelines.xhtml

For peer review only

before 37 weeks gestation, while 83.06% (N=361,300) were born at term or later (37 weeks and onwards).

The Born in Wales database has recently been utilised to investigate the risk factors associated with low birth weight (LBW), defined as a birth weight of less than 2500g. Analyses revealed that non-singleton children exhibited the highest risk of LBW (OR 21.74 (95% CI 21.09 to 22.40)), followed by pregnancy intervals of less than 1 year (OR 2.92 (95% CI 2.70 to 3.15)) [24]. Maternal physical and mental health conditions, including diabetes (OR 2.03 (95% CI 1.81 to 2.28)), anaemia (OR 1.26 (95% CI 1.16 to 1.36)), depression (OR 1.58 (95% CI 1.43 to 1.75)), serious mental illness (OR 1.46 (95% CI 1.04 to 2.05)), anxiety (OR 1.22 (95% CI 1.08 to 1.38)) and use of antidepressant medication during pregnancy (OR 1.92 (95% CI 1.20 to 3.07)) were also identified as significant risk factors for LBW. Additional maternal risk factors include smoking (OR 1.80 (95% CI 1.76 to 1.84)), alcohol-related hospital admission (OR 1.60 (95% CI 1.30 to 1.97)), substance misuse (OR 1.35 (95% CI 1.29 to 1.41)) and evidence of domestic abuse (OR 1.98 (95% CI 1.39 to 2.81)) [24]. Numbers and percentages are provided in the supplementary information.

Born in Wales survey participants

2,500 parents have completed the Born in Wales surveys. The mean age of Born in Wales survey participants was 32 years (SD=12.5), with an interquartile range (IQR) of 28-35 years. The Born in Wales survey data was utilised in a mixed methods study conducted during the COVID-19 pandemic, aiming to examine the effects of the pandemic on pregnancy experiences and birth outcomes in 2020. Results indicated that the pandemic had a notable adverse effect on the psychological wellbeing of 71% of survey respondents. These individuals reported heightened

For peer review only - http://bmjopen.bmj.com/site/about/guidelines.xhtml

levels of anxiety, stress, and feelings of loneliness, which were associated with attending prenatal scans in the absence of their partner, giving birth alone, and restricted interactions with midwives. However, no significant differences were observed in annual birth outcomes, including gestation, birth weight, stillbirths, and Caesarean sections, between infants born in 2020 compared to 2016–2019. Whilst the pandemic negatively affected mothers' pregnancy experiences, population-level data indicates that this did not result in adverse birth outcomes for infants born during the pandemic [25].

HAPPEN children's primary school network

The Health & Attainment of Pupils in Primary EducatioN (HAPPEN) network represents a substantial cohort of over 30,000 school-aged children across Wales, with a gender distribution of approximately 47% boys, 49% girls, and 3% who preferred not to disclose their gender. The average age of the children is 9.35 years. Recently, the HAPPEN dataset has been utilised to explore the health and wellbeing of children during the COVID-19 pandemic. This encompassed a retrospective cohort study employing an online cohort survey conducted between January 2018 to February 2020, in conjunction with routine PCR SARS-CoV-2 test results. The study explored health-related behaviours in children spanning the period from 2018 to 2020, and their association with being tested and testing positive in 2020 to 2021. Notably, the investigation revealed significant associations between parental health literacy and monitoring behaviours [26]. Furthermore, the study employed free school meal status (FSM) as a proxy for deprivation in an exploratory analysis examining the impact of school closures on the health and wellbeing. The findings indicated that children eligible for FSM may experience adverse consequences in terms of physical health, including reduced physical activity and suboptimal dietary choices, as a result of prolonged school closures [27].

For peer review only - http://bmjopen.bmj.com/site/about/guidelines.xhtml

Discussion

Strengths and limitations

Born in Wales has established a comprehensive, Wales-wide population-based database which consolidates clinical data from maternity, neonatal, child health, and education records. This national-scale database is supplemented by quantitative and qualitative results from surveys conducted by Born in Wales, providing rich insights into details that cannot be obtained through routinely-collected data. The existence of this database enables further data linkage, facilitating life course research on the health and wellbeing of the Wales population. This has significant implications for enhancing healthcare delivery at the local level and offering valuable research evidence to guide policy and practise both within the studied population and in other countries. This research aims to generate robust evidence that can influence policy and practice, enabling the implementation of early interventions to promote child health and wellbeing. Moreover, collaborations with diverse cohorts facilitate comparisons across different populations, enabling a broader understanding of factors influencing child health and wellbeing outcomes.

One inherent constraint associated with routine and administrative data is the presence of missing data or errors. However, SAIL has established a robust framework that includes a team of skilled analysts and rigorous quality control procedures to address issues such as duplication of patient data entries and minimise instances of missing data. Subsequently, the Born in Wales project can contribute to the enhancement of clinical reporting practice and bolster the reliability of research findings derived from the database. Additionally, the utilisation of anonymised cohorts serves as an effective strategy for overcoming the barriers related to obtaining consent from individuals, thereby facilitating the seamless aggregation and analysis of data.

For peer review only - http://bmjopen.bmj.com/site/about/guidelines.xhtml

A potential constraint of Born in Wales is the loss of data pertaining to individuals who relocate outside of Wales during pregnancy or after the child's birth. Analogous to other datasets, research conducted utilising Born in Wales may be constrained by the information available through routine data collection. Nevertheless, Born in Wales can enhance the available information by incorporating survey questions that capture data missing from administrative records. It is important to acknowledge that data entry can be subject to human error, and there may still be instances of missing records.

Research aspirations

As the Born in Wales database continues to expand, there are intentions to broaden the scope by incorporating additional health, social care, and education data, alongside Police data. The subsequent phase of data linkage will involve integrating Police data into SAIL. Replicating this data linkage model on a national scale within the UK and potentially extending it globally would enable the establishment of more extensive research cohorts and facilitate cross-comparisons across diverse populations.

This cohort is established through the integration of routine data records, encompassing health and administrative data, as well as environmental data supplemented with survey data to enrich the cohort. It constitutes a comprehensive, data rich total population cohort constructed through substantial investments and extensive record linkage efforts from HDR UK and ADR. Furthermore, this methodology demonstrates cost-effectiveness by leveraging existing data sources, establishing a dynamic cohort that continuously expands its participant pool. The cohort is well suited for investigating natural experiments and interventions. The endeavours undertaken

For peer review only - http://bmjopen.bmj.com/site/about/guidelines.xhtml

in this study closely align with recommendations 1-3 and 6 of the MRC strategy to maximise UK population cohorts [28].

The design of this cohort facilitates cross-cohort comparisons with other electronic cohorts in the UK, including Born in Bradford [3] and eLIXIR [4]. To harmonise phenotypic variables across studies and adopt core common data standards, all data in the cohort will adhere to the Observational Medical Outcomes Partnership (OMOP) standard. Knowledge sharing and directories have been facilitated through the HDR UK gateway, and high-quality, robust meta-data is provided, adhering to recommendations 4 and 5 of the MRC strategy [28].

This cohort is unique due to the comprehensive breadth of routine and survey data collected and linked in a total population cohort. It is designed to enable data sharing by employing harmonised data and meta-data, following the principles of findability, accessibility, interoperability, and reusability (FAIR). A distinguishing feature of Born in Wales is its incorporation of census data linked to numerous datasets, encompassing social care and justice datasets. Additionally, the database includes self-reported data which captures aspects that are not typically electronically captured, such as stress, wellbeing, physical activity, access to services, and use of services.

Data availability and access

The data for the Born in Wales cohort is available in the SAIL Databank at Swansea University, Swansea, UK [29]. All proposals to use SAIL data are subject to review by an independent Information Governance Review Panel (IGRP). This project's approval code is 0916. Before any data can be accessed, approval must be given by the IGRP. To use Born in Wales data you need

For peer review only - http://bmjopen.bmj.com/site/about/guidelines.xhtml

to provide a safe researcher training certificate, a signed data access agreement and IGRP approval.

Future directions

A comprehensive research database has been established, encompassing data pertaining to maternity, neonatal, and child health. This database integrates information on maternal and paternal health throughout pregnancy and extends to encompass the subsequent health of the child. Moreover, it enriches the dataset with valuable qualitative insights obtained from the Born in Wales surveys, which capture nuanced information not captured solely by hospital records. Born in Wales is committed to continuous progress and expansion through collaborative efforts, enabling longitudinal follow-up of families in Wales and fostering co-production of research questions relevant to each stage of development. By encompassing the entire population of Wales, Born in Wales generates a wealth of rich data on the national scale, which has the potential to inform interventions aimed at promoting healthy growth and development among the population.

For peer review only

Data availability statement: Data are available upon reasonable request. Researchers can apply for data access by submitting a research application to the SAIL team. The SAIL website provides information on the application process (https://saildatabank.com/data/apply-to-work-with-the-data/). All proposals to use SAIL data are subject to review by an independent Information Governance Review Panel (IGRP). This project's approval code is 0916. Before any data can be accessed, approval must be given by the IGRP. To use Born in Wales data you need to provide a safe researcher training certificate, a signed data access agreement and IGRP approval.

Funding: This work was supported by the National Centre for Population Health and Wellbeing Research (NCPHWR) grant number [AMS103836].

Contributors: All authors provided substantial contributions to the piece of work. Jones, Brophy, and Dredge contributed to the conception of this article. Seaborne, Kennedy, James, Bandyopadhyay, Battaglia, and Davies were involved in manuscript writing and revision. Jones, Seaborne, Bandyopadhyay, and James were involved in data analysis and interpretation. All authors read and approved the final manuscript. All authors agree to be accountable for all aspects of the work.

For peer review only

References

[1] O'Connor M, Spry E, Patton G et al. Better together: Advancing life course research through multi-cohort analytic approaches. *Adv. Life Course Res*. 2022;53:100499.

[2] Mikkelsen B, Williams J, Rakovac I et al. Life course approach to prevention and control of non-communicable diseases. *BMJ* [Internet]. 2019;364:l257. Available from: https://www.bmj.com/content/364/bmj.l257

[3] Wright J, Small N, Raynor P et al. Cohort Profile: The Born in Bradford multi-ethnic family cohort study. *Int. J. Epidemiol.* [Internet]. 2012 Oct 12 [cited 2019];42(4):978–91. Available from: https://academic.oup.com/ije/article/42/4/978/655870

[4] Carson LE, Azmi B, Jewell A et al. Cohort profile: the eLIXIR Partnership—a maternity–child data linkage for life course research in South London, UK. *BMJ Open*. 2020;10(10):e039583.

[5] Born in Scotland [Internet]. The University of Edinburgh. 2022 [cited 2023 Jun 14]. Available from: https://www.ed.ac.uk/cardiovascular-science/born-in-scotland

[6] Li M, Wang Z, Zhang B et al. Sample attrition analysis in a prospective cohort study of medical graduates in China. *BMC Med. Res. Methodol.* 2022;22(1).

[7] Ferri-García R, Rueda M del M. Propensity score adjustment using machine learning classification algorithms to control selection bias in online surveys. Marchetti S, editor. *PLoS One*. 2020;15(4):e0231500.

[8] Our Research [Internet]. HDR UK. Available from: https://www.hdruk.ac.uk/research/

For peer review only - http://bmjopen.bmj.com/site/about/guidelines.xhtml

[9] Gray L. The importance of post hoc approaches for overcoming non-response and attrition bias in population-sampled studies. *Soc. Psychiatry Psychiatr. Epidemiol*. 2015;51(1):155–7.

[10] Todberg T, Koch A, Andersson M et al. Incidence of Otitis Media in a Contemporary Danish National Birth Cohort. Schooling CM, editor. *PLoS One*. 2014;9(12):e111732.

[11] Brown AS, Sourander A, Hinkka-Yli-Salomäki S et al . Elevated maternal C-reactive protein and autism in a national birth cohort. *Mol. Psychiatry* [Internet]. 2014 Feb 1 [cited 2022];19(2):259–64. Available from: https://pubmed.ncbi.nlm.nih.gov/23337946/

[12] Norman JE, Morris C, Chalmers JD. The Effect of Changing Patterns of Obstetric Care in Scotland (1980–2004) on Rates of Preterm Birth and Its Neonatal Consequences: Perinatal Database Study. *PLoS Med*. 2009;6(9):e1000153–3.

[13] Martin P, Cortina-Borja M, Newburn M et al. Timing of singleton births by onset of labour and mode of birth in NHS maternity units in England, 2005–2014: A study of linked birth registration, birth notification, and hospital episode data. Dangal G, editor. *PLoS One*. 2018;13(6):e0198183.

[14] Thornton CE, Dahlen HG. Born before arrival in NSW, Australia (2000–2011): a linked population data study of incidence, location, associated factors and maternal and neonatal outcomes. *BMJ Open* [Internet]. 2018;8(3):e019328. Available from: https://bmjopen.bmj.com/content/bmjopen/8/3/e019328.full.pdf

[15] Harper G. Linkage of Maternity Hospital Episode Statistics data to birth registration and notification records for births in England 2005–2014: Quality assurance of linkage of routine data for singleton and multiple births. *BMJ Open*. 2018;8(3):e017898.

For peer review only - http://bmjopen.bmj.com/site/about/guidelines.xhtml

[16] Dattani N, Macfarlane A. Linkage of Maternity Hospital Episode Statistics data to birth registration and notification records for births in England 2005-2014: methods. A population-based birth cohort study. *BMJ Open*. 2018;8(2):e017897.

[17] Birth cohort data linkage study [Internet]. ADR UK. [cited 2023 Jun 14]. Available from: https://www.adruk.org/our-mission/our-impact/article/birth-cohort-data-linkage-study-228/

[18] Jones KH, Ford DV, Thompson S et al. A Profile of the SAIL Databank on the UK Secure Research Platform. *IJPDS*. 2020;4(2).

[19] Lyons RA, Ford DV, Moore L et al. Use of data linkage to measure the population health effect of non-health-care interventions. *Lancet*. 2014;383(9927):1517–9.

[20] Born in Wales [Internet]. NCPHWR. [cited 2023 Jun 14]. Available from: https://ncphwr.org.uk/portfolio/born-in-wales-overview/

[21] CORDS (Co-production of Research Direction and Strategy) [Internet]. NCPHWR. [cited 2023 Jun 14]. Available from: https://ncphwr.org.uk/portfolio/cords-co-production/

[22] Guidance on co-producing a research project – INVOLVE [Internet]. Available from: https://www.invo.org.uk/posttypepublication/guidance-on-co-producing-a-research-project/

[23] Public Involvement in Research Impact Toolkit (PIRIT) [Internet]. Cardiff University. Available from: https://www.cardiff.ac.uk/marie-curie-research-centre/patient-and-public-involvement/public-involvement-in-research-impact-toolkit-pirit

[24] Bandyopadhyay A, Jones H, Parker M et al. Weighting of risk factors for low birth weight: a linked routine data cohort study in Wales, UK. *BMJ Open*. 2023;13(2):e063836–6.

For peer review only - http://bmjopen.bmj.com/site/about/guidelines.xhtml

[25] Jones H, Seaborne M, Cowley L et al. Population birth outcomes in 2020 and experiences of expectant mothers during the COVID-19 pandemic: A "born in Wales" mixed methods study using routine data. Magnus MC, editor. *PLoS One*. 2022;17(5):e0267176

[26] Marchant E, Lowthian E, Crick T et al. Pre-COVID-19 pandemic health-related behaviours in children (2018–2020) and association with being tested for SARS-CoV-2 and testing positive for SARS-CoV-2 (2020–2021): a retrospective cohort study using survey data linked with routine health data in Wales, UK. *BMJ Open*. 2022;12(9):e061344–4.

[27] James M, Marchant E, Defeyter MA et al. Impact of school closures on the health and well-being of primary school children in Wales UK: a routine data linkage study using the HAPPEN Survey (2018–2020). *BMJ Open*. 2021;11(10):e051574.

[28] Maximising the value of UK population cohorts MRC strategic Review of the largest UK Population cohort studies [Internet]. [cited 2023 Jun 14]. Available from: https://www.ukri.org/wp-content/uploads/2021/12/MRC-23122021-UK-Population-cohorts.pdf

[29] Ford DV, Jones KH, Verplancke JP, et al. The SAIL Databank: building a national architecture for e-health research and evaluation. *BMC Health Serv. Res*. 2009;9(1)

For peer review only

For peer review only - http://bmjopen.bmj.com/site/about/guidelines.xhtml

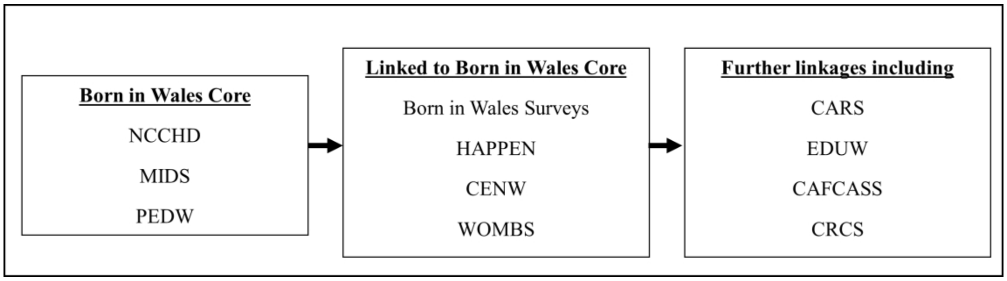

Figure 1. Core components of Born in Wales and further linkages

434x118mm (157 x 157 DPI)

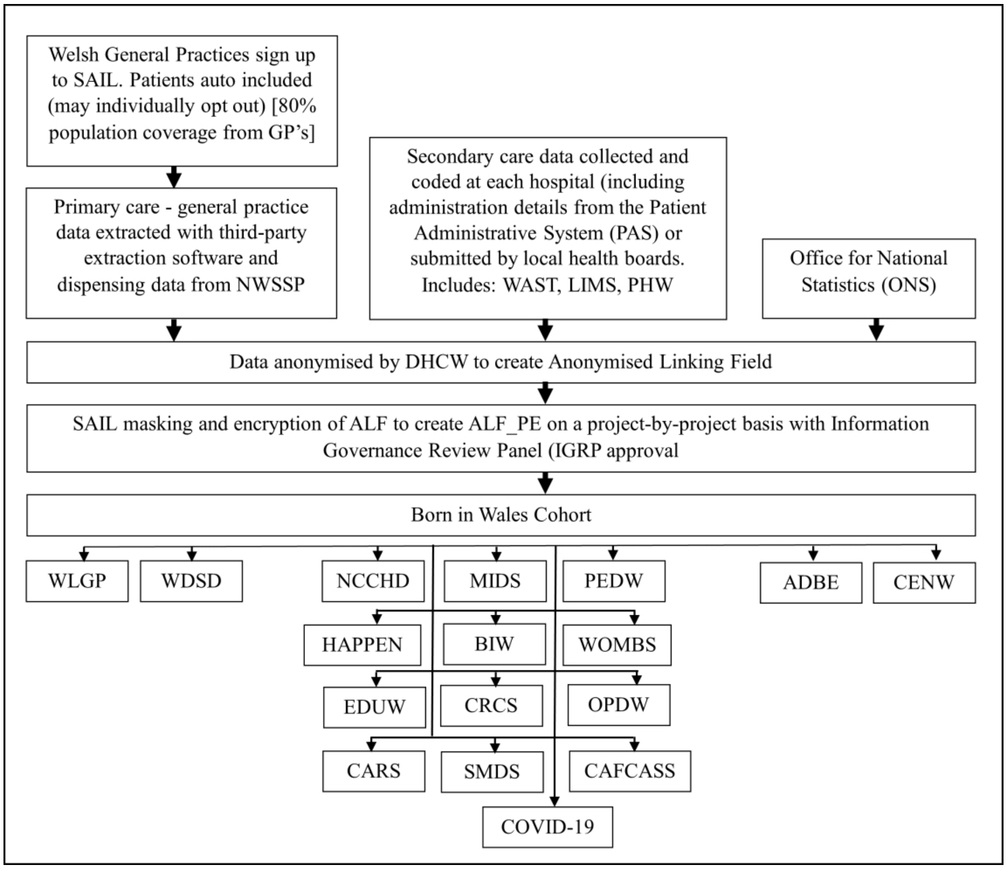

Figure 2. Flow diagram of the SAIL process

429x368mm (157 x 157 DPI)

Supplementary Information

Variable	N	%
**Pregnancy Interval**		
Only/first child	345,645	49.85
Less than 1 year	6,234	0.90
1-2 years	73,319	10.57
2-5 years	176,109	25.40
5-7 years	45,141	6.51
7-10 years	29,868	4.31
Above 10 years	17,061	2.46
**Diabetes**		
No	692,777	99.91
Yes	600	0.09
**Anaemia**		
No	673,912	97.19
Yes	19,465	2.81
**Depression**		
No	684,553	98.73
Yes	8,824	1.27
**Serious mental illness**		
No	693,072	99.96
Yes	305	0.04
**Anxiety**		
No	682,812	98.48
Yes	10,565	1.52
**Antridepressant medication**		
No	693,213	99.98
Yes	164	0.02
**Smoking**		
No	544,258	78.49
Yes	149,119	21.51
**Alcohol-related hospital admission**		
No	692,709	99.90
Yes	668	0.10
**Substance misuse**		
No	658,520	94.97
Yes	34,857	5.03
**Domestic abuse**		
No	692,644	99.89
Yes	733	0.11

For peer review only

STROBE 2007 (v4) Statement—Checklist of items that should be included in reports of *cohort studies*

Section/Topic	Item #	Recommendation	Reported on page #
**Title and abstract**	1	(*a*) Indicate the study's design with a commonly used term in the title or the abstract	0
		(*b*) Provide in the abstract an informative and balanced summary of what was done and what was found	2
**Introduction**			
Background/rationale	2	Explain the scientific background and rationale for the investigation being reported	5-7
Objectives	3	State specific objectives, including any prespecified hypotheses	7
**Methods**			
Study design	4	Present key elements of study design early in the paper	8-9
Setting	5	Describe the setting, locations, and relevant dates, including periods of recruitment, exposure, follow-up, and data collection	8
Participants	6	(*a*) Give the eligibility criteria, and the sources and methods of selection of participants. Describe methods of follow-up	8-9
		(*b*) For matched studies, give matching criteria and number of exposed and unexposed	NA
Variables	7	Clearly define all outcomes, exposures, predictors, potential confounders, and effect modifiers. Give diagnostic criteria, if applicable	NA
Data sources/ measurement	8*	For each variable of interest, give sources of data and details of methods of assessment (measurement). Describe comparability of assessment methods if there is more than one group	9-11
Bias	9	Describe any efforts to address potential sources of bias	12
Study size	10	Explain how the study size was arrived at	15
Quantitative variables	11	Explain how quantitative variables were handled in the analyses. If applicable, describe which groupings were chosen and why	15
Statistical methods	12	(*a*) Describe all statistical methods, including those used to control for confounding	NA
		(*b*) Describe any methods used to examine subgroups and interactions	17-19
		(*c*) Explain how missing data were addressed	NA
		(*d*) If applicable, explain how loss to follow-up was addressed	NA
		(*e*) Describe any sensitivity analyses	NA
**Results**			

For peer review only

Participants	13*	(a) Report numbers of individuals at each stage of study—eg numbers potentially eligible, examined for eligibility, confirmed eligible, included in the study, completing follow-up, and analysed	NA
		(b) Give reasons for non-participation at each stage	NA
		(c) Consider use of a flow diagram	12
Descriptive data	14*	(a) Give characteristics of study participants (eg demographic, clinical, social) and information on exposures and potential confounders	15-19
		(b) Indicate number of participants with missing data for each variable of interest	15-17
		(c) Summarise follow-up time (eg, average and total amount)	15
Outcome data	15*	Report numbers of outcome events or summary measures over time	17-19
Main results	16	(*a*) Give unadjusted estimates and, if applicable, confounder-adjusted estimates and their precision (eg, 95% confidence interval). Make clear which confounders were adjusted for and why they were included	NA
		(*b*) Report category boundaries when continuous variables were categorized	NA
		(*c*) If relevant, consider translating estimates of relative risk into absolute risk for a meaningful time period	NA
Other analyses	17	Report other analyses done—eg analyses of subgroups and interactions, and sensitivity analyses	17-19
**Discussion**			
Key results	18	Summarise key results with reference to study objectives	20
**Limitations**			
Interpretation	20	Give a cautious overall interpretation of results considering objectives, limitations, multiplicity of analyses, results from similar studies, and other relevant evidence	20
Generalisability	21	Discuss the generalisability (external validity) of the study results	23
**Other information**			
Funding	22	Give the source of funding and the role of the funders for the present study and, if applicable, for the original study on which the present article is based	24

*Give information separately for cases and controls in case-control studies and, if applicable, for exposed and unexposed groups in cohort and cross-sectional studies.

Note: An Explanation and Elaboration article discusses each checklist item and gives methodological background and published examples of transparent reporting. The STROBE checklist is best used in conjunction with this article (freely available on the Web sites of PLoS Medicine at http://www.plosmedicine.org/, Annals of Internal Medicine at http://www.annals.org/, and Epidemiology at http://www.epidem.com/). Information on the STROBE Initiative is available at www.strobe-statement.org.

For peer review only
